# Impacted Third Molars, A Rare Occurrence of Identical Bilateral Impacted Mandibular Third Molars in Linguo-Buccal Location: A Case Report

**DOI:** 10.7759/cureus.20858

**Published:** 2021-12-31

**Authors:** Shaul Hameed Kolarkodi, Fatimah M Alharbi, Reema Aljurbua, Lina aloufi, Hessah almubarak

**Affiliations:** 1 Maxillofacial Surgery and Diagnostic Science, Qassim University, Qassim, SAU; 2 Dentistry, Qassim University, Qassim, SAU

**Keywords:** surgical extraction, lingo-buccal direction, symmetrical, bilateral, impaction

## Abstract

Symmetrical horizontal impacted bilateral mandibular third molar in lingo-buccal direction is a rare type of impacted teeth. When a tooth cannot erupt and fails to achieve a normal function and occlusion during its chronological age of eruption, it is called an impacted tooth. In this paper, a case of a male patient aged 20 years old who had bilateral horizontally impacted lower third molar which was noticed in a routine screening panoramic radiograph and confirmed with cone beam computed tomography (CBCT) imaging and referred to the oral maxillofacial surgical center for surgical removal of the impacted teeth in order to avoid late complications is discussed.

## Introduction

Impacted teeth are a phenomenon when the tooth cannot erupt into its proper position. It may be completely or partially erupted [1,2]. In a normal situation, it erupts at ages ranging from 16 to 24 years (mean: 20) [3,4]. The most commonly impacted teeth are mandibular and maxillary third molars followed by an upper canine, and they may remain undetected because they are usually asymptomatic [5].

Local factors causing third molar impaction may include crowding or supernumerary teeth or may be associated with various pathologies lesions such as dentigerous cysts, calcifying odontogenic cysts, unicystic (mural) ameloblastomas, ameloblastomas, ameloblastic fibromas, adenomatoid odontogenic tumors, keratocystic odontogenic tumors, calcifying epithelial odontogenic tumors, ameloblastic fibro-odontomas, and odontomas [6].

We can assess the impacted molars by using Pell and Gregory classification which depends on the depth level of the impacted tooth according to their relationship to the occlusal surface of the adjacent second molar [7,8]. Wafa classified impacted teeth depending on the inclination of the crown of an impacted tooth to the long axis of the second molar [9]. Radiological examinations are considered important to determine the complications during and after the surgery [6].

Our case is associated with a rare type of impaction. It is bilaterally impacted of the mandibular third molar which transverse in a lingo-buccal, horizontal orientation in a symmetric pattern, and it is classified by Pell and Gregory as type Class III B (due to complete lingual deflection).

## Case presentation

**Figure 1:**
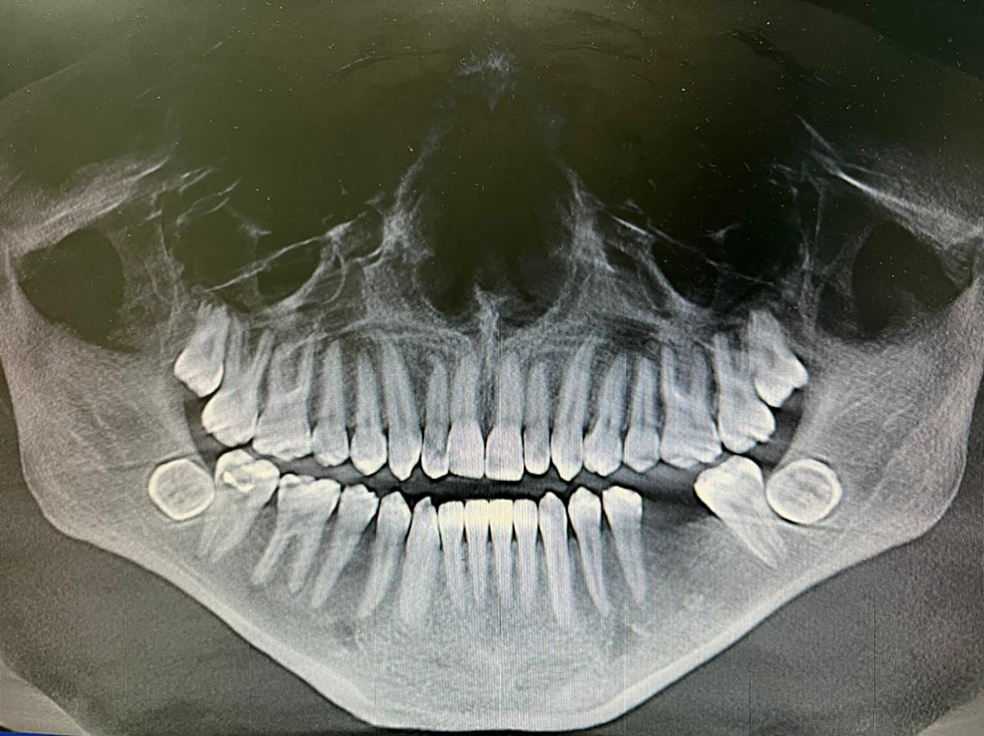
CBCT panoramic view showed a bilateral impacted mandibular third molars CBCT, Cone-beam computed tomography.

A panoramic screening radiograph showed a bilateral impacted mandibular third molar which was transversed in a lingo-buccal direction (Figure 1). Upon clinical extra-oral examination, the face was symmetrical without any lesions. However, intraoral examination revealed that the buccal and lingual cortical bones were slightly expanded on palpation, and there were no pathological signs or symptoms.

**Figure 2:**
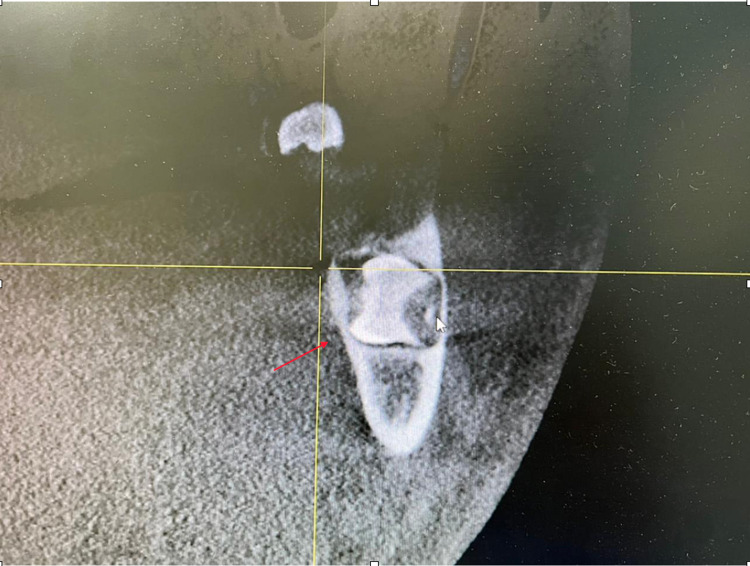
CBCT of the sagittal slice showing thinning on lingual cortical plates CBCT, Cone beam computed tomography.

**Figure 3:**
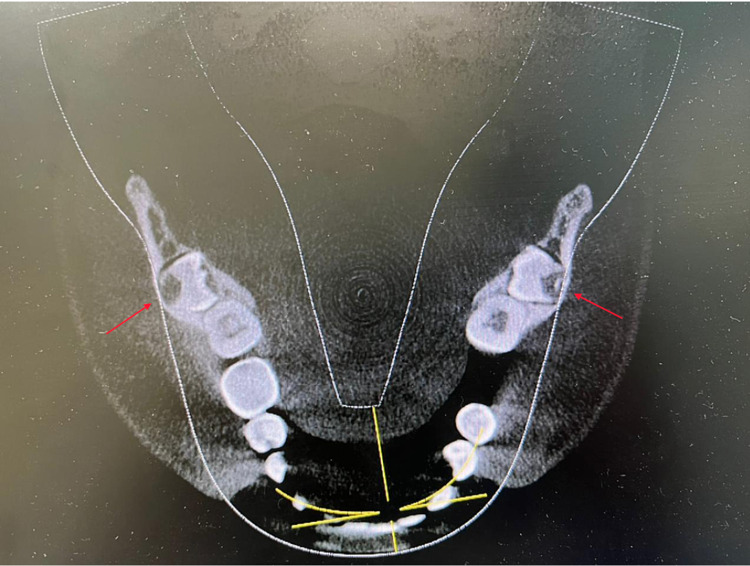
CBCT of the axial section showing the root of 38-48 not completely formed and the impacted teeth in horizontally placed in lingo-buccal direction CBCT, Cone beam computed tomography.

**Figure 4:**
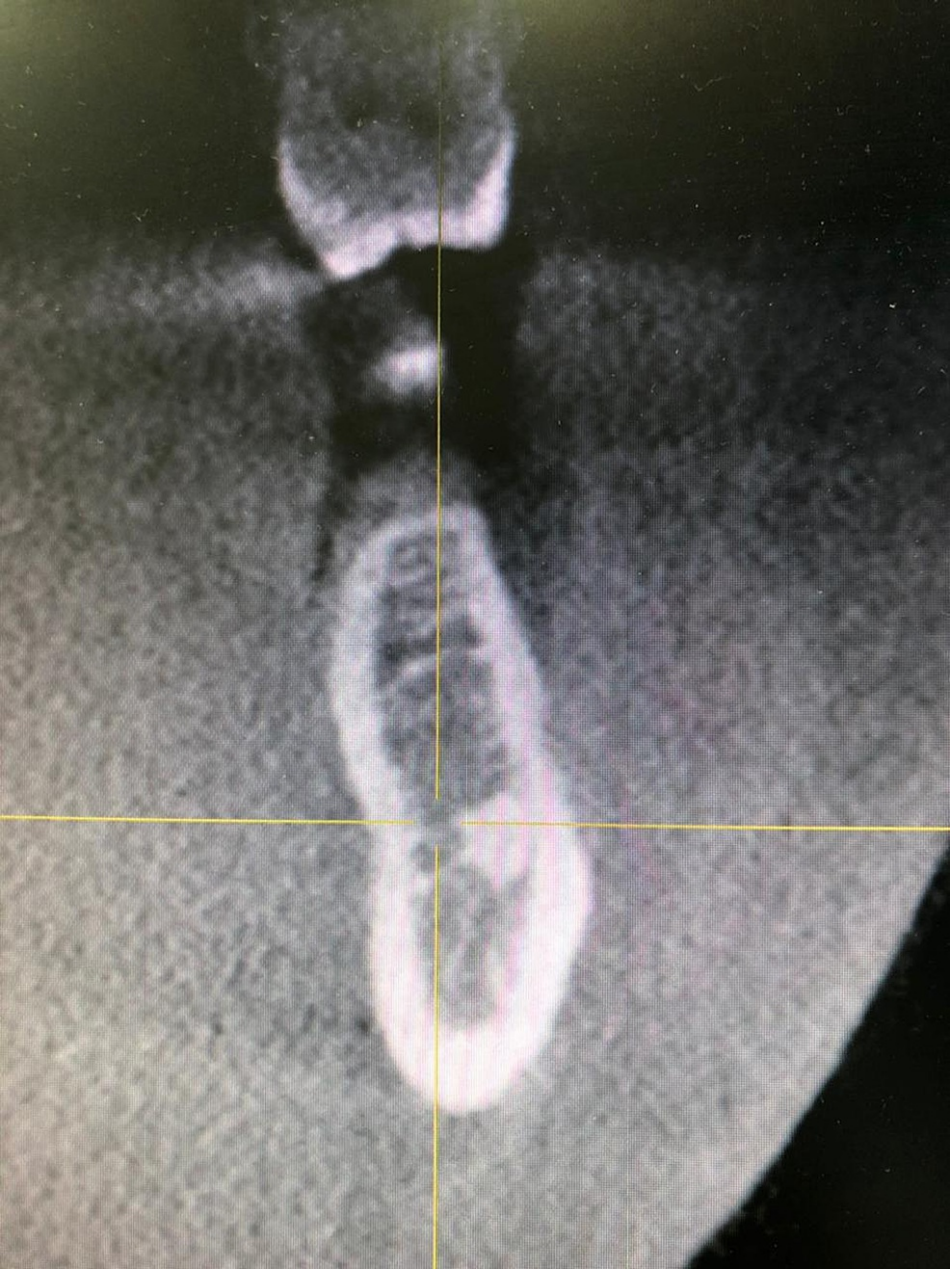
CBCT of the coronal slice showing the idiopathic osteosclerosis CBCT, Cone beam computed tomography.

**Figure 5:**
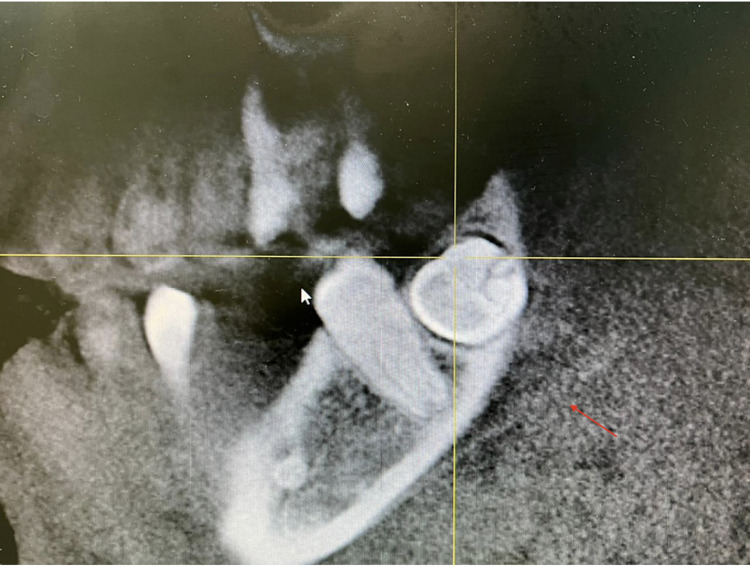
CBCT of the coronal slice showing the relationship between horizontal impacted and inferior alveolar canal CBCT, Cone beam computed tomography.

**Figure 6:**
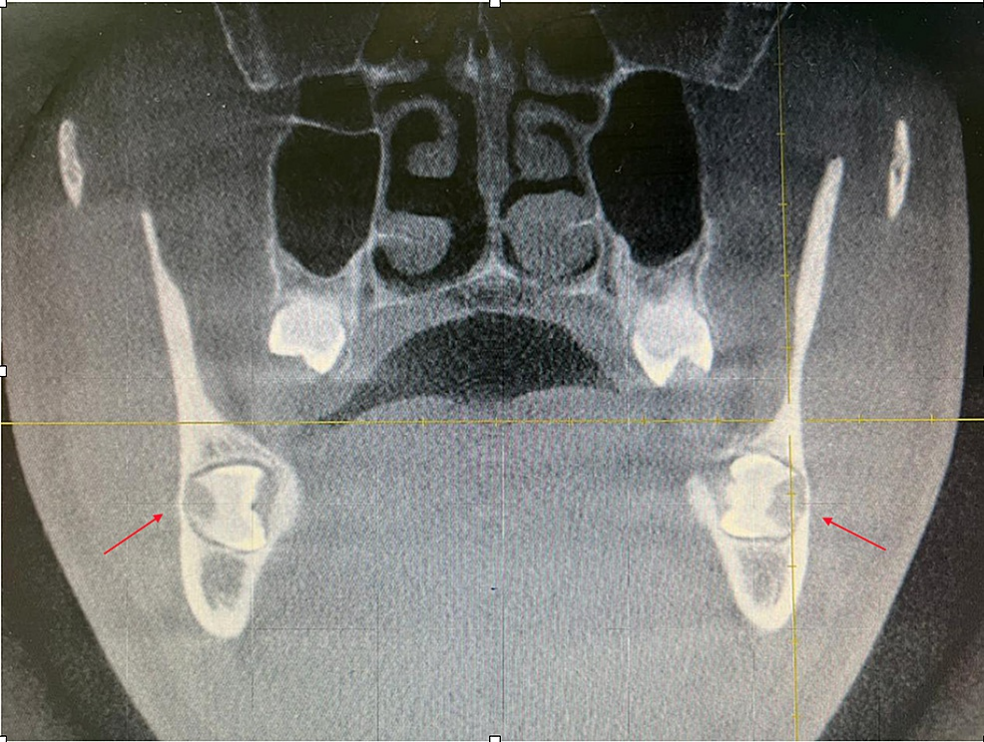
CBCT of the sagittal slice showing bilateral symmetrically impacted teeth CBCT, Cone beam computed tomography.

**Figure 7:**
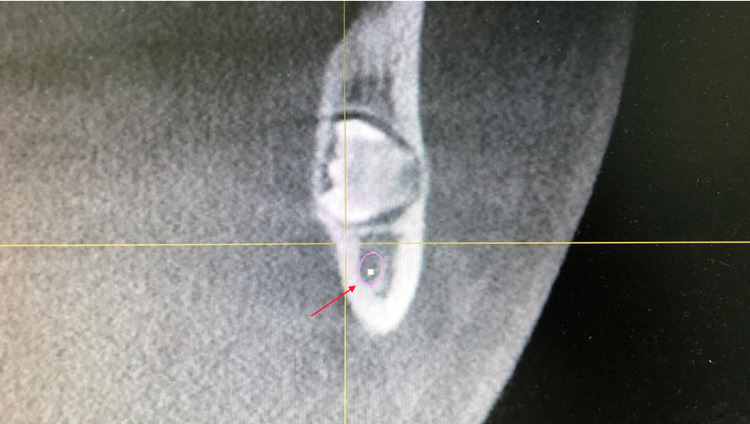
CBCT of the coronal slice showing the proximity of impacted tooth to the lingual nerve CBCT, Cone beam computed tomography.

After informing the patient about the condition of his impacted mandibular third molars, a further investigation was needed. Cone beam computed tomography (CBCT) was requested for a proper evaluation. The CBCT images revealed that the third molars were positioned in a linguo-buccal direction on both sides, with a noted thinning on the lingual cortical plates. There were no pathological findings associated with the molars impacted (Figure 2). After a detailed evaluation of the CBCT results, the root of impacted teeth was not completely formed and also revealed idiopathic osteosclerosis (Figures 3-7) in relation to the second premolar region. The treatment options were discussed with the patient, and the benefits and risks were clearly explained. The patient's treatment of choice was to extract his impacted third molars; thereafter, he was referred to a higher center to receive the needed treatment.

## Discussion

The most common impacted are mandibular third molars and maxillary third molars because of the lack of space in the dental or due to pathological change that may obstruct the normal eruption of the third molar [10,11]. Transverse bilateral symmetrical impactions are a form of impaction that is very rarely reported in dental literature [12], especially symmetrical linguoverted impacted bilateral lower third molar, a horizontal impaction that orient in buccolingual direction with crown overlap root [13].

In this case, the crowns of the teeth face the lingual cortical plate and the roots toward the buccal cortical plate, and the root formation is not complete, The decision for extraction of asymptomatic impactions represents a surgical dilemma. The impacted tooth is the source of infection by harbor bacteria and results in pericoronitis in soft tissue or odontogenic cyst and resorption in hard tissue. The impacted teeth have a higher incidence of dentigerous cysts than unicystic ameloblastoma and odontogenic keratocyst. These cysts' dental remnants surround the fully impacted teeth as well-defined pericoronal radiolucency on x-ray [13,14].

The risk of injury of nerves during the removal of impacted third molars in surgery, especially the lingual nerve, during an increase in the buccolingual position of the lower third molars due to the anatomical proximity, lack of accessibility, and proximity to lingual nerve rather than inferior alveolar nerve due to linguo-buccal location of impacted tooth [15,16] and surgery of unerupted impacted lower third molars was reported as a more surgical complication compared with erupted teeth [16,17].

However, a study by Blondeau and Daniel revealed interesting reports that the postoperative complications increase with patient age. Therefore, they recommend immediate extraction of the impacted mandibular third molar, once a decision has been made, well before the age of 25 years especially for women as they had significantly more problems with alveolitis, infections, and paresthesia than men. No specific factor was identified to explain this difference between the sexes [18].

The patient was informed about the postoperative complications that include swelling, pain, trismus, localized alveolar osteitis (alveolitis sicca dolorosa), infection, and lingual nerve paresthesia resulting in numbness on the lateral half of the tongue/IAN paresthesia. There are many theories as to the cause of alveolar osteitis. The two most common causes for dry socket are clot lysis and the development of alveolar osteitis in the presence of tissue or bacterial activated fibrinolysis [19].

## Conclusions

In dental practice, clinicians encounter various types of impaction of teeth, among which symmetrical horizontal impacted bilateral lower third molar in lingo-buccal direction is a rare type of impaction. Two treatment options were described in the scientific literature review, which were follow-up asymptomatic impaction or surgical extraction of all impacted teeth, and we highly recommend surgical removal of both impacted teeth at either side of the mandible in order to avoid late complications.
